# Endoplasmic reticulum-associated protein degradation contributes to Toll innate immune defense in *Drosophila melanogaster*

**DOI:** 10.3389/fimmu.2022.1099637

**Published:** 2023-01-19

**Authors:** Yangyang Zhu, Lei Liu, Chuchu Zhang, Chao Zhang, Tingting Han, Renjie Duan, Yiheng Jin, Huimin Guo, Kan She, Yihua Xiao, Akira Goto, Qingshuang Cai, Shanming Ji

**Affiliations:** ^1^ Center for Developmental Biology, School of Life Sciences, Anhui Agricultural University, Hefei, Anhui, China; ^2^ School of Preclinical Medicine, Wannan Medical College, Wuhu, Anhui, China; ^3^ Center for Biological Technology, Anhui Agricultural University, Hefei, Anhui, China; ^4^ Université de Strasbourg, Centre National de la Recherche Scientifique (CNRS), Insect Models of Innate Immunity (M3I; UPR9022), Strasbourg, France; ^5^ Institut de Génétique et de Biologie Moléculaire et Cellulaire, Illkirch, France

**Keywords:** Sip3, Me31B, Xbp1, ERAD, Toll innate immune defense, *Drosophila melanogaster*

## Abstract

In *Drosophila*, the endoplasmic reticulum-associated protein degradation (ERAD) is engaged in regulating pleiotropic biological processes, with regard to retinal degeneration, intestinal homeostasis, and organismal development. The extent to which it functions in controlling the fly innate immune defense, however, remains largely unknown. Here, we show that blockade of the ERAD in fat bodies antagonizes the Toll but not the IMD innate immune defense in *Drosophila*. Genetic approaches further suggest a functional role of Me31B in the ERAD-mediated fly innate immunity. Moreover, we provide evidence that silence of *Xbp1* other than *PERK* or *Atf6* partially rescues the immune defects by the dysregulated ERAD in fat bodies. Collectively, our study uncovers an essential function of the ERAD in mediating the Toll innate immune reaction in *Drosophila*.

## Introduction

Innate immunity is the first line of the defense for the host against invasion with foreign pathogenic microorganisms (1, 2). Upon activation of the pattern recognition receptors (PRRs) through sensing distinct pathogen-associated molecular patterns (PAMPs), a series of innate immune signaling pathways entail complex downstream cascades to evoke the synthesis of multiple pro-inflammatory cytokines contributing to defeating the invading pathogens (1–4). Up to date, a large body of pioneering studies have highlighted the importance of the precise control of these signals, as defective or over-excessive innate immune response routinely trigger the occurrences of autoimmune disease, allergic reaction, and even cancer (5–9). Unraveling the molecular mechanisms of how innate immune signaling pathways are dynamically modulated has always been one of the hotspots in the basic immunology research.

Over the last decades, *Drosophila melanogaster* has served as one of the fundamental animal models for studying the regulatory mechanisms of the host innate immune reaction. In *Drosophila*, the innate immune defense is mainly dominated by two classical nuclear factor-kappa B (NF-κB)-related signaling pathways, namely the Toll and the immune deficiency (IMD) pathways, which rely on their productions of various antimicrobial peptides (AMPs) to function as anti-infective agents (10, 11). The *Drosophila* Toll pathway receptors are normally activated following infection of fungi or some types of Gram-positive bacteria. This activation further induces signal transductions involving factors including Myd88, pelle, as well as tube, and ultimately the phosphorylation of cactus, releasing the transcription factor Dif/dorsal into the nucleus to direct the production of a series of AMPs, such as Drosomycin (Drs) and Metchnikowin (Mtk) (12, 13). The IMD signaling pathway is typically activated post challenging of most Gram-negative bacteria or some species of Gram-positive bacteria, controlling the expression of an alternative set of AMPs such as Attacin (Att) and Diptericin (Dpt) (12, 14). In particular, both signaling pathways have been demonstrated to be precisely controlled by a large body of modulators. For instance, ubiquitination modification plays a pivotal role in Imd signaling transduction and leveling off (15–25), thus contributing to its homeostasis. While ubiquitin-involved cascade in the Toll pathway mediation was previously thought to be relatively rare, recent findings have been shedding light on this issue (26, 27).

In an effort to uncover novel regulators of the fly Toll pathway, we adopted the widely-used *drosomycin* promoter-luciferase (Drs-Luc) reporter system and performed an unbiased screening of various genes in *Drosophila* S2 cells. Of interest, we found that silence of *Septin-interacting protein 3* (*Sip3*, also known as *Hrd1*) resulted in marked reduction in the Toll signaling activities (**Figure S1A**), implying that *Sip3* is potentially a positive contributor of the Toll pathway. Previous literature has revealed that Sip3 is a multiplexed transmembrane protein in the endoplasmic reticulum (ER) behaving as an E3 ubiquitin ligase (28). The predominant function of Sip3 is to catalyze the degradation of misfolded/unfolded proteins in the ER *via* specifically accepting ubiquitin from the ER-associated E2 enzyme and transferring it to the substrate to facilitate its degradation (known as ER-associated degradation, ERAD) (28, 29). We therefore hypothesized that the Sip3-involved ERAD is probably essential for the Toll-mediated innate immune defense in *Drosophila*.

In this study, we demonstrate a critical role of the ERAD in regulating fly innate immunity. We show that blockade of the ERAD cascade in *Drosophila* fat bodies results in reduced inductions of the Toll downstream AMPs upon Gram-positive bacterial challenging. We further provide a series of evidence that the ERAD is required for the host resistance against various pathogenic microbes. Additionally, our proteomic and genetic results imply that the dysregulated ERAD leads to abnormal accumulation of Me31B, which may further negatively contribute to the Toll innate immune defense primarily in an Xbp1-dependent manner. Overall, our studies shed lights on an essential role of the ERAD in modulating the Toll innate immunity in *Drosophila*.

## Results

### Sip3 is engaged in regulating the Toll innate immune response in *Drosophila*

**Figure 1:**
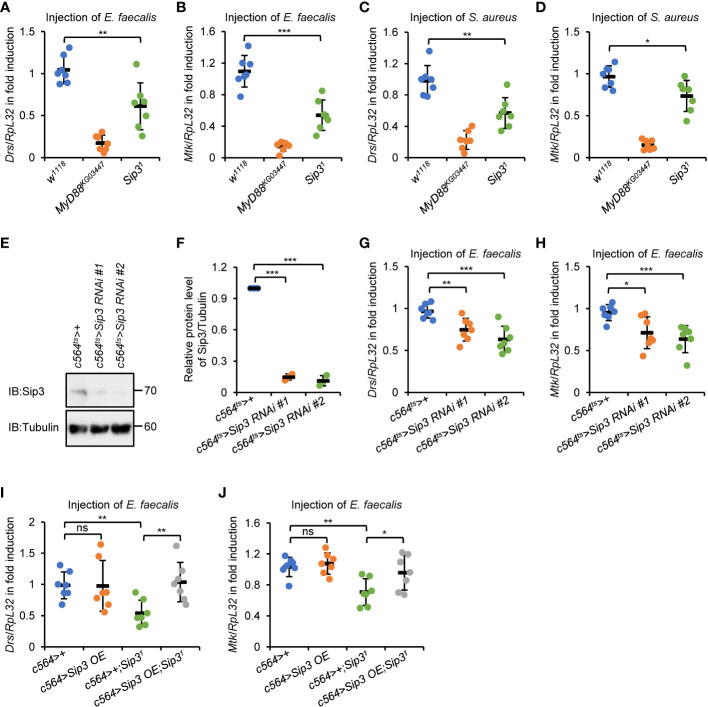
E3 ligase Sip3 positively regulates Toll signaling in *Drosophila*. **(A–D)** *Wild-type* (*w^1118^*), *MyD88^KG03447^*, and *Sip3^1^* mutant flies were injected with *E. faecalis* **(A, B)** or *S. aureus* **(C, D)** for 12 h, followed by RT-qPCR assays to monitor the expression levels of *Drs* **(A, C)** or *Mtk* **(B, D)**. Each dot represents one independent replicate (10 flies for each replicate). **(E, F)** Fat bodies were dissected from flies (10 flies for each sample) including *c564^ts^>+*, *c564^ts^>Sip3 RNAi #1*, and *c564^ts^>Sip3 RNAi #2*, followed by Western blot assays **(E)**. Tubulin was used as loading control. Densitometry analysis to quantify the protein level of samples in **(E)** is shown in **(F)**. **(G, H)** Flies including *c564^ts^>+*, *c564^ts^>Sip3 RNAi #1*, and *c564^ts^>Sip3 RNAi #2* were infected with *E. faecalis.* 12* h* later, flies were harvested for RT-qPCR assays to detect the transcript levels of *Drs* **(G)** or *Mtk* **(H)**. Each dot represents one independent replicate (10 flies for each replicate). **(I)** and **(J)** Flies including *c564>+*, *c564>Sip3 OE*, *c564>+;Sip3^1^*, and *c564>Sip3OE;Sip3^1^* were infected with *E. faecalis*, followed by RT-qPCR assays as in **(G)** and **(H)**. Each dot represents one independent replicate (10 flies for each replicate). In **(A–D)** and **(F, J)**, data are shown as mean ± standard errors. *p < 0.05; **p < 0.01; ***p < 0.001; ns, not significant.

In order to investigate whether the E3 ligase Sip3 is involved in controlling *Drosophila* Toll innate immune response *in vivo*, we infected *Sip3* loss-of-function (LOF) mutant flies (*Sip3^1^*, isogenized with *w^1118^*) and the controls (*w^1118^* as the *wild-type* control and *MyD88^KG03447^* homozygote as the positive control) with *Enterococcus faecalis* (*E. faecalis*), a type of Gram-positive bacteria that has been widely utilized to activate the Toll signaling pathway in flies. The reverse transcription plus quantitative polymerase chain reaction (RT-qPCR) assays were performed to monitor the transcript levels of the Toll downstream AMPs, including *Drs* and *Mtk*. As illustrated in **Figures 1A, B**, loss of *Sip3* resulted in marked decreases in the mRNA levels of both *Drs* (by ~42%) and *Mtk* (by ~51%) upon bacterial challenging, which is consistent with what we found in S2 cells (**Figure S1A**). Of note, similar results were obtained (**Figures 1C, D**) when flies were challenged with another type of Gram-positive bacteria *Staphylococcus aureus* (*S. aureus*).

To test whether Sip3 modulates Toll signaling through its potential role in the fat body, which is the dominant immune organ/tissue of flies during systemic infection, we adopted two different *Sip3 RNAi* strains (see Materials and Methods) and crossed them with *c564-gal4* to produce progenies with fat body-specific down-regulation of *Sip3*. The *tub-gal80^ts^* was utilized in our experimental system to carry out RNAi of *Sip3* at adult stage. The RNAi efficiency in those *Sip3 RNAi* progenies (referred as *c564^ts^>Sip3 RNAi #1* and *c564^ts^>Sip3 RNAi #2*) was tested by Western blot using anti-Sip3 antibody (**Figures 1E, F**). Further, these flies were infected with *E. faecalis* and subjected to RT-qPCR as described above. As illustrated in **Figures 1G, H**, silencing *Sip3* reduced the transcript levels of both *Drs* (by ~23% to 35%) and *Mtk* (by ~25% to 28%) relative to those in the controls (*c564^ts^>+*). Moreover, ectopic expression of *Sip3* in the fat body reversed the reductions in *Drs* and *Mtk* transcript levels by loss of *Sip3* upon infection (**Figures 1I, J**), implying that the E3 ligase Sip3 controls the activation of Toll signaling post bacterial stimuli primarily through its essential role in the fat body. Notably, we failed to observe any apparent alterations in the context of pathogen-driven inductions of the Toll downstream AMPs in the *c564>Sip3* flies, compared to those in the controls (**Figures 1I, J**).

**Figure 2:**
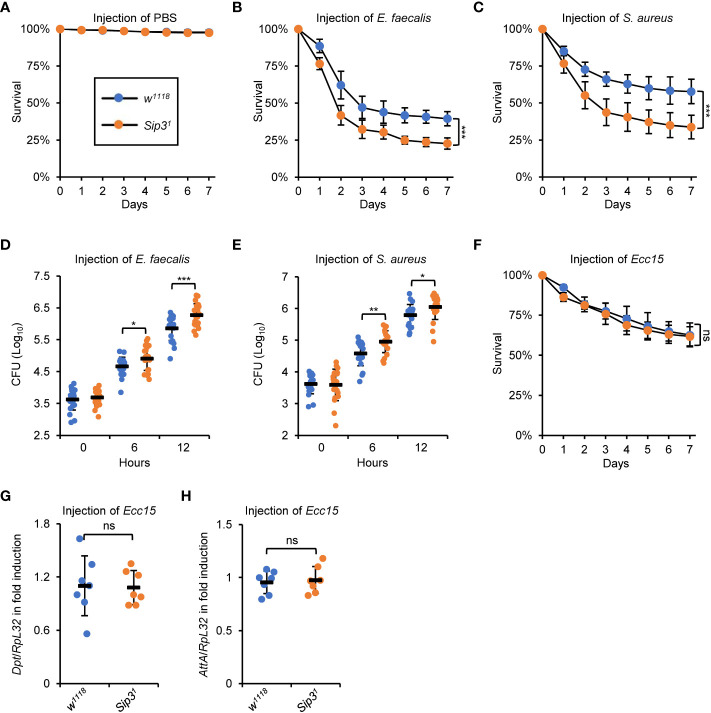
Sip3 contributes to the host resistance against injected bacteria. **(A–C)** *Wild-type* and *Sip3* mutant flies were infected with sterile PBS **(A)**, *E. faecalis* **(B)**, or *S. aureus* **(C)**. Flies were then counted for mortality every day. The numbers of flies analyzed in A, B and C were as follows. In **(A)**, *WT*: 104, 105, 105; *Sip3*: 105, 102, 107. In **(B)**, *WT*: 107, 106, 103; *Sip3*: 104, 107, 105. In **(C)**, *WT*: 105, 107, 103; *Sip3*: 104, 102, 106. **(D, E)** *Wild-type* and *Sip3* mutant flies were infected with *E. faecalis* **(D)**, or *S. aureus* **(E)**. At indicated time points (0, 6, and 12 h), flies were subjected to bacterial burden assays. Each dot represents one independent replicate (10 flies for each replicate). **(F–H)** *Wild-type* and *Sip3* mutant flies were infected with *Ecc15*, followed by survival rate assays **(F)** or RT-qPCR assays **(G, H)**. In **(F)**, the numbers of flies were as follows. *WT*: 104, 107, 105; *Sip3*: 105, 102, 107. In **(G)** and **(H)**, each dot represents one independent replicate (10 flies for each replicate). All the data were shown as mean ± standard errors. *p < 0.05; **p < 0.01; ***p < 0.001; ns, not significant.

A detailed understanding of the physiological function of Sip3 in regulating the host innate immune defense was achieved by analyzing the fly survival post infection of *E. faecalis* or *S. aureus*, as *Sip3* LOF mutants were more susceptible to both pathogens than the *wild-type* flies (**Figures 2A–C**). Further, we quantified the amounts of bacteria present in the flies at various time points post infection (0, 6, and 12h, respectively). Much higher levels of colony-forming units (CFU) were observed in the samples of *Sip3* LOF mutants (increased on average by more than 1-fold), relative to those in the control samples (**Figures 2D, E**), implying that *Sip3* defective flies are less capable of scavenging pathogenic microorganisms. Similar results were obtained when we performed infection experiments with *E. faecalis* utilizing *Sip3 RNAi* and the control flies (**Figures S2A, B**).

In summary, our results indicate that E3 ligase Sip3 plays a crucial role in the Toll innate immune defense in fruit flies.

### Sip3 is dispensable for impacting the IMD immune reaction in *Drosophila*

In *Drosophila*, the humoral immune defense is principally governed by two signaling pathways, namely the Toll and the IMD pathways (11). It is thus reasonable to explore whether Sip3 is as well involved in regulating the fly IMD innate immunity. To this end, an alternative set of infection experiment was conducted utilizing the *Erwinia carotovora carotovora 15* (*Ecc15*), a type of broadly-used Gram-negative bacteria activating the IMD pathway in flies. Unfortunately, we hardly detected apparent alterations in the contexts of host survival (**Figure 2F**) and IMD downstream AMP inductions (**Figures 2G, H**), between those of the *Sip3* LOF mutants and the *wild-type* controls upon *Ecc15* injection. Collectively, our data endorse the notion that Sip3 is required for the Toll but not the IMD innate immune reaction in *Drosophila*.

### ERAD is essential for *Drosophila* Toll innate immune defense

**Figure 3:**
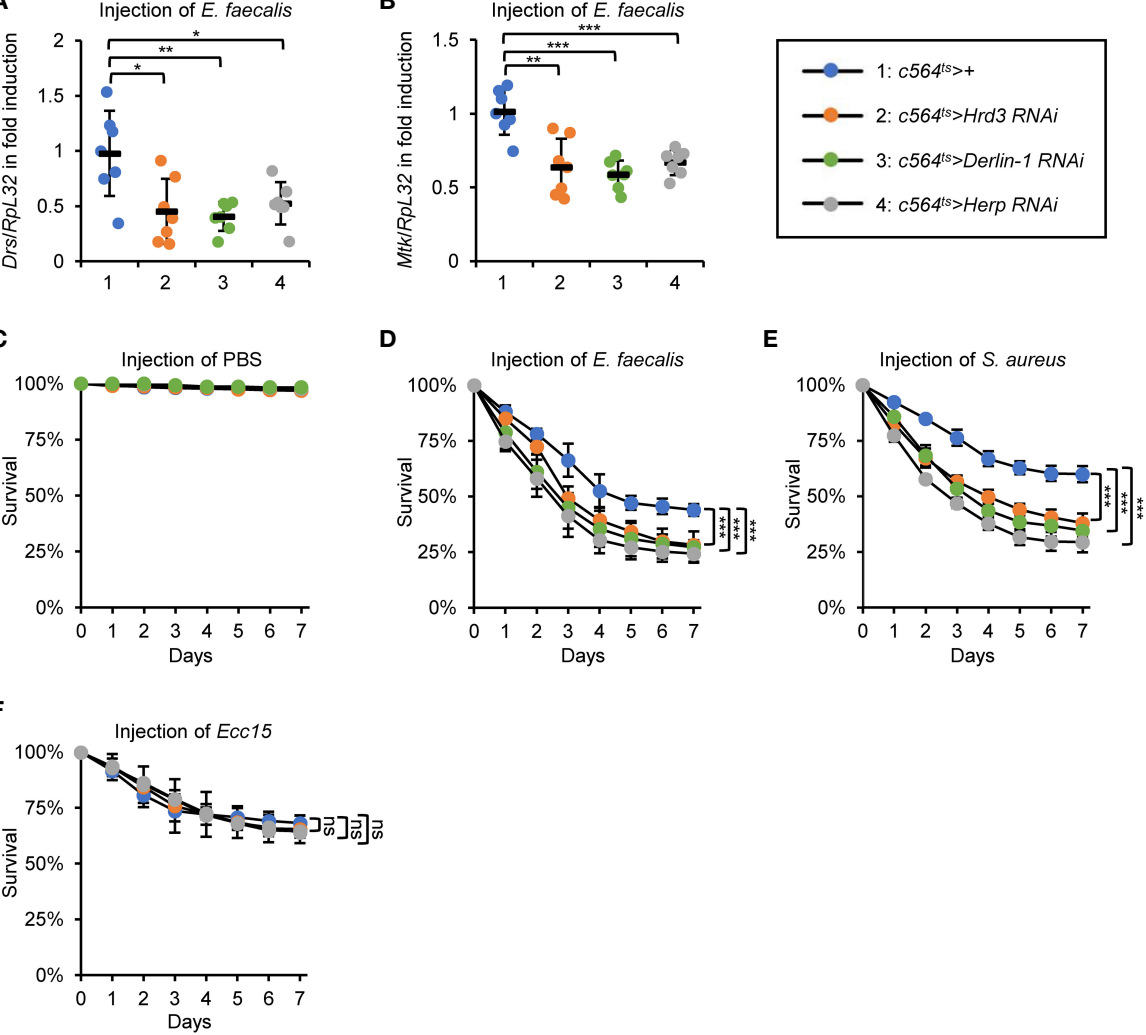
ERAD is essential for the fly Toll innate immune defense. **(A, B)** Flies including 1: *c564^ts^>+*, 2: *c564^ts^>Hrd3 RNAi*, 3: *c564^ts^>Derlin-1 RNAi*, and 4: *c564^ts^>Herp RNAi* were infected with *E. faecalis.* 12* h* later, flies were harvested for RT-qPCR assays to detect the mRNA levels of *Drs* **(A)** or *Mtk* **(B)**. Each dot represents one independent replicate (10 flies for each replicate). **(C–F)** Flies including 1: *c564^ts^>+*, 2: *c564^ts^>Hrd3 RNAi*, 3: *c564^ts^>Derlin-1 RNAi*, and 4: *c564^ts^>Herp RNAi* were injected with sterile PBS **(C)**, *E. faecalis* **(D)**, *S. aureus* **(E)**, or *Ecc15* **(F)**, followed by survival rate assays. The numbers of flies were as follows. In **(C)**, *c564^ts^>+*: 107, 106, 106; *c564^ts^>Hrd3 RNAi*: 102, 107, 105; *c564^ts^>Derlin-1 RNAi*: 103, 104, 107; *c564^ts^>Herp RNAi*: 103, 102, 102. In **(D)**, *c564^ts^>+*: 104, 103, 105; *c564^ts^>Hrd3 RNAi*: 105, 106, 104; *c564^ts^>Derlin-1 RNAi*: 107, 105, 104; *c564^ts^>Herp RNAi*: 105, 102, 106. In **(E)**, *c564^ts^>+*: 106, 104, 107; *c564^ts^>Hrd3 RNAi*: 105, 106, 105; *c564^ts^>Derlin-1 RNAi*: 102, 103, 105; *c564^ts^>Herp RNAi*: 104, 104, 102. In **(F)**, *c564^ts^>+*: 104, 103, 104; *c564^ts^>Hrd3 RNAi*: 104, 105, 107; *c564^ts^>Derlin-1 RNAi*: 106, 102, 103; *c564^ts^>Herp RNAi*: 103, 105, 105. All the data were shown as mean ± standard errors. *p < 0.05; **p < 0.01; ***p < 0.001; ns, not significant.

As mentioned in the Introduction, Sip3 has been demonstrated to be responsible for the degradation of misfolded/unfolded proteins accumulated in the ER following a range of stresses (for instance metabolic dysfunction, excessive accumulation of reactive oxygen species, infection, etc.), a process known as ERAD (28, 29). We then sought to investigate whether other components of the ERAD also participate in affecting fly innate immunity, which is analogous to Sip3. To do this, we again debited the *c564^ts^* system to obtain flies with fat body-specific RNAi of *Hrd3*, *Derlin-1*, or *Herp*, which encode pivotal elements of the fly ERAD cascade (28, 30). As shown in **Figures 3A, B**, silence of *Hrd3*, *Derlin-1*, or *Herp* resulted in marked abatements (by ~34% to 58%) in the inductions of *Drs* and *Mtk* post bacterial stimuli. Accordingly, the *c564^ts^* >*Hrd3 RNAi*, *c564^ts^* >*Derlin-1 RNAi*, and *c564^ts^* >*Herp RNAi* flies were more sensitive to the injected *E. faecalis* and *S. aureus* (**Figures 3C–E**) but not *Ecc15* (**Figure 3F**) with respective to the controls. Taken together, our results support the notion that the ERAD is responsible for the *Drosophila* Toll innate immune defense upon bacterial infection.

### ERAD regulates Toll-mediated innate immune response largely *via* Me31B

**Figure 4:**
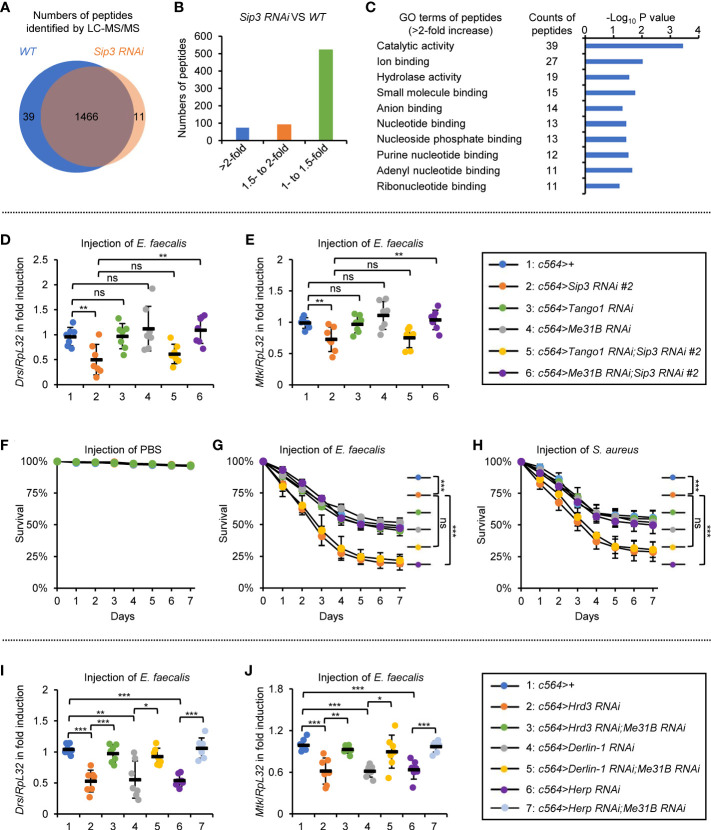
ERAD modulates Toll signaling in an Me31B-dependent manner. **(A–C)** Proteomic analysis of fat bodies dissected from *wild-type* (*c564^ts^*) and *Sip3 RNAi* (*c564^ts^>Sip3 RNAi 2#*) flies (100 flies for each sample, 3 biological replicate for each group). In **(A)**, total numbers of peptides/proteins that were identified by the LC-MS/MS assay. In **(B)**, numbers of peptides that were increased by various indicated folds (>2-fold, 1.5- to 2-fold, or 1- to 1.5-fold) in the *Sip3 RNAi* samples relative to the controls. In **(C)**, gene ontology analysis of the increased (>2-fold) peptides/proteins. **(D, E)** Flies including 1: *c564>+*, 2: *c564>Sip3 RNAi #2*, 3: *c564>Tango1 RNAi*, 4: *c564>Me31B RNAi*, 5: *c564>Tango1 RNAi;Sip3 RNAi #2*, and 6: *c564>Me31B RNAi;Sip3 RNAi #2* were infected with *E. faecalis*. 12 h later, flies were subjected to RT-qPCR assays. Each dot represents one independent replicate (10 flies for each replicate). **(F–H)** Flies with same genotypes as in **(D)** were injected with sterile PBS **(F)**, *E. faecalis* **(G)**, or *S. aureus* **(H)**, followed by survival rate assays. The numbers of flies were as follows. In **(F)**, *c564>+*:105, 103, 102; *c564>Sip3 RNAi #2*: 104, 105, 106; *c564>Tango1 RNAi*: 105, 106, 105; *c564>Me31B RNAi*: 102, 103, 105; *c564>Tango1 RNAi;Sip3 RNAi #2*: 106, 104, 107; *c564>Me31B RNAi;Sip3 RNAi #2*: 106, 104, 102. In **(G)**, *c564>+*:103, 104, 107; *c564>Sip3 RNAi #2*: 103, 105, 103; *c564>Tango1 RNAi*: 105, 102, 103; *c564>Me31B RNAi*: 106, 106, 106; *c564>Tango1 RNAi;Sip3 RNAi #2*: 104, 106, 105; *c564>Me31B RNAi;Sip3 RNAi #2*: 106, 102, 104. In **(H)**, *c564>+*:101, 105, 104; *c564>Sip3 RNAi #2*: 104, 104, 104; *c564>Tango1 RNAi*: 104, 102, 105; *c564>Me31B RNAi*: 103, 106, 102; *c564>Tango1 RNAi;Sip3 RNAi #2*: 106, 104, 103; *c564>Me31B RNAi;Sip3 RNAi #2*: 102, 107, 106. **(I)** and **(J)** Flies including 1: *c564>+*, 2: *c564>Hrd3 RNAi*, 3: *c564>Hrd3 RNAi;Me31B RNAi*, 4: *c564>Derlin-1 RNAi*, 5: *c564>Derlin-1 RNAi;Me31B RNAi*, 6: *c564>Herp RNAi*, and 7: *c564>Herp RNAi;Me31B RNAi* were infected with *E. faecalis*. 12 h later, flies were subjected to RT-qPCR assays. Each dot represents one independent replicate (10 flies for each replicate). All the data were shown as mean ± standard errors. *p < 0.05; **p < 0.01; ***p < 0.001; ns, not significant.

We next sought to address how the ERAD is implicated in impacting the innate immune defense in *Drosophila*. A proteomic analysis was performed using dissected fat bodies from *c564^ts^>Sip3 RNAi* and the control flies following bacterial injection (**Supplementary Table 1**). Whereas over 1500 proteins/peptides were identified in this assay, the majority (more than 95%) of them overlapped in both samples (**Figure 4A**), implying that the E3 ligase Sip3 is of low potential for silencing or evoking gene expression. The fact that knockdown of *Sip3* in S2 cells certainly leads to a reduction in Toll signaling activity (**Figure S1A**) makes us assume that when *Sip3* is silenced, one (or possibly more) pivotal factors of the Toll pathway is dysregulated. However, we failed to observe any significant differences when we compared the appearances of protein/peptide of Myd88, pll, tube, cactus, dorsal, and Dif from both samples (**Figure S3A**), even though the Toll downstream effectors (e.g. BaraA2, Drs, Mtk, IM1) were markedly reduced by *Sip3 RNAi* (**Figure S3B**). Thus, we reasoned that the Sip3-mediated ERAD likely engage in an indirect way to modulate the fly Toll pathway.

Since blockade of the ERAD leads to accumulation of misfolded/unfolded proteins, we thus prioritized higher existences of proteins/peptides present in the *Sip3 RNAi* flies, as we hypothesized that they are probably the key to the deregulated Toll signaling. Based upon the data from the proteomic analysis, only around 70 proteins/peptides out of the ~1500 were significantly increased (by more than 2-fold) by down-regulation of *Sip3* (**Figure 4B**). Gene ontology (GO) analysis upon the increased proteins/peptides indicated that the top 3 categories regarding their molecular functions were 1) catalytical activity, 2) ion binding, and 3) hydrolase activity (**Figure 4C**).

We first focused on the top 10 increased proteins/peptides (including Cbp80, Kat60, Arts, Tango1, Cpb, LManV, Me31B, Vasa, CG7900, and CG31974). They were barely detectable in the control samples, whereas they enormously accumulated in the *Sip3 RNAi* fat bodies. Among these, Tango1 and Me31B are the only two candidates considered to be ER-related proteins (31, 32), allowing us to postulate that they are likely under the control of the ERAD and play potential roles in the ERAD-mediated innate immunity of *Drosophila*. To test this assumption, we used *Tango1 RNAi* and *Me31B RNAi* flies for genetic manipulations. We observed that the induction of *Drs* or *Mtk* post bacterial infection was not affected by knockdown of *Tango1* or *Me31B* in fat bodies (**Figures 4D, E**). However, double knock down of *Me31B* and *Sip3* restored the induction of the Toll pathway-regulated AMPs, and this was not observed in the case of *Tango1* and *Sip3* double RNAi (**Figures 4D, E**). Pioneering studies have demonstrated that *Drosophila* Me31B is a putative DEAD-box helicase, which is functionally involved in protein translation and mRNA decay through a set of associations with other adaptor proteins (33–37). Consistently, when we looked at the fly survival post infection of *E. faecalis* or *S. aureus*, we found that the low resistances to bacterial infection in *Sip3 RNAi* flies were profoundly elevated by *Me31B RNAi* (**Figures 4F–H**). Moreover, loss of *Me31B* also prevented the passive impacts on bacteria-driven AMP inductions by RNAi of *Hrd3*, *Derlin-1*, or *Herp* (**Figures 4I, J**). Taken together, our results indicate that the ERAD contributes to the Toll innate immune defense to a large extent in an *Me31B*-dependent manner in *Drosophila*.

### Silence of *Xbp1* partially rescues the phenotype of *Sip3* mutants

**Figure 5:**
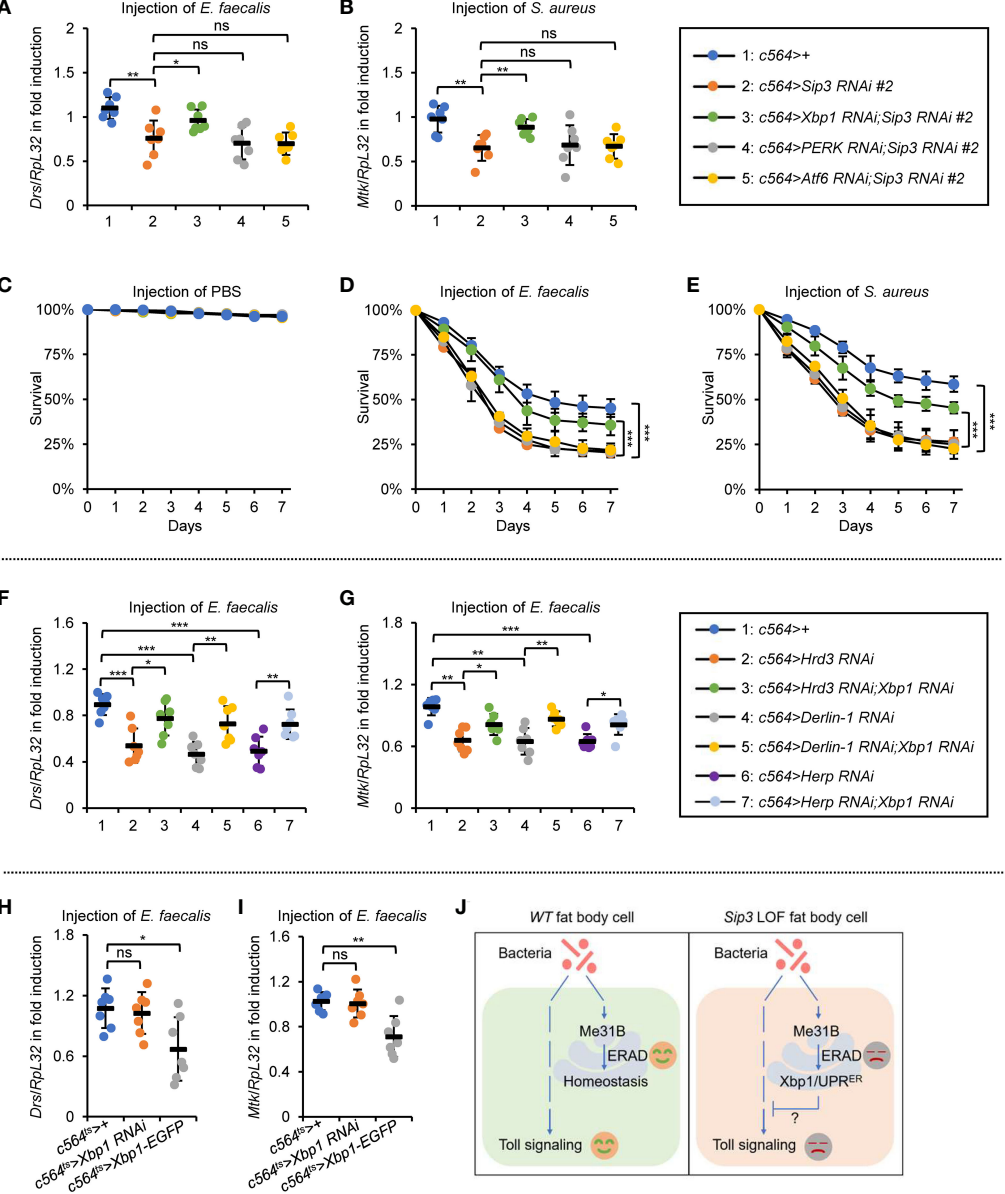
ERAD controls the Toll innate immunity partially *via* the Xbp1 axis of UPR^ER^. **(A, B)** Flies including 1: *c564>+*, 2: *c564>Sip3 RNAi #2*, 3: *c564>Xbp1 RNAi;Sip3 RNAi #2*, 4: *c564>PERK RNAi;Sip3 RNAi #2*, and 5: *c564>Atf6 RNAi;Sip3 RNAi #2* were infected with *E. faecalis*, followed by RT-qPCR assays. Each dot represents one independent replicate (10 flies for each replicate). **(C–E)** Flies with same genotypes as in **(A)** were injected with sterile PBS **(C)**, *E. faecalis* **(D)**, or *S. aureus* **(E)**, followed by survival rate assays. The numbers of flies were as follows. In **(C)**, *c564>+*: 106, 102, 104; *c564>Sip3 RNAi #2*: 104, 106, 103; *c564>Xbp1 RNAi;Sip3 RNAi #2*: 103, 105, 103; *c564>PERK RNAi;Sip3 RNAi #2*: 103, 105, 106; *c564>Atf6 RNAi;Sip3 RNAi #2*: 102, 107, 102. In **(D)**, *c564>+*: 103, 105, 103; *c564>Sip3 RNAi #2*: 104, 106, 106; *c564>Xbp1 RNAi;Sip3 RNAi #2*: 107, 105, 105; *c564>PERK RNAi;Sip3 RNAi #2*: 104, 102, 103; *c564>Atf6 RNAi;Sip3 RNAi #2*: 105, 102, 102. In **(E)**, *c564>+*: 102, 104, 103; *c564>Sip3 RNAi #2*: 106, 105, 107; *c564>Xbp1 RNAi;Sip3 RNAi #2*: 105, 102, 104; *c564>PERK RNAi;Sip3 RNAi #2*: 106, 106, 101; *c564>Atf6 RNAi;Sip3 RNAi #2*: 103, 104, 105. **(F)** and **(G)** Flies including 1: *c564>+*, 2: *c564>Hrd3 RNAi #2*, 3: *c564>Hrd3 RNAi;Xbp1 RNAi*, 4: *c564>Derlin-1 RNAi*, 5: *c564>Derlin-1 RNAi;Xbp1 RNAi*, 6: *c564>Herp RNAi*, and 7: *c564>Herp RNAi;Xbp1 RNAi* were infected with *E. faecalis*, followed by RT-qPCR assays. Each dot represents one independent replicate (10 flies for each replicate). **(H)** and **(I)** Flies including *c564^ts^>+*, *c564^ts^>Xbp1 RNAi*, and *c564^ts^>Xbp1-EGFP* were infected with *E. faecalis*. RT-qPCR assays were performed to monitor the transcript levels of *Drs* **(H)** and *Mtk* **(I)**. Each dot represents one independent replicate (10 flies for each replicate). **(J)** Working model by which ERAD modulates *Drosophila* Toll innate immune response. In the *wild-type* (*WT*) fat bodies, the ERAD remains homeostasis without negatively impacting Toll signaling. When ERAD is blocked (e.g. in the *Sip3* LOF fat bodies), accumulated Me31B activates the Xbp1 axis of the UPR^ER^, thereby antagonizing the Toll pathway. In **(A–I)**, data were shown as mean ± standard errors. *p < 0.05; **p < 0.01; ***p < 0.001; ns, not significant.

Our next aim is to explore how accumulation of Me31B negatively impacts on Toll signaling. It is reported that the accumulated misfolded/unfolded proteins on ER can be recognized by diverse stress sensors, namely inositol acquisition enzyme-1 (IRE1), double-stranded RNA-activated protein kinase-like ER kinase (PERK), and/or activated transcription factor 6 (ATF6), in a manner that leads to the activation of specific signaling cascades (referred to as unfolded protein response to ER, UPR^ER^) (29, 38–40). We thereby sought to determine whether the stockpiled Me31B by the ERAD dysfunction is responsible for the activation of UPR^ER^, which passively impacts on the Toll innate immune pathway. Indeed, we observed an elevated mRNA occurrence of *Xbp1RB* (generated by splicing of *Xbp1* mRNA) and *PERK* in the *Sip3 RNAi* fat bodies (**Figures S4A, B**), implicating that activation of UPR^ER^ in the fly fat bodies with the ERAD blockade. Notably, we detected decreased transcript levels of both *Drs* and *Mtk* in the *Sip3 RNAi* samples relative to those in the control (**Figures S4C, D**). In addition, we obtained flies with fat body-specific RNAi of *Sip3* combined with *Xbp1*, *PERK*, or *Atf6 RNAi* and conducted infection experiments. As illustrated in **Figures 5A, B**, down regulation of *PERK* or *Atf6* was dispensable for affecting the *Sip3 RNAi*-mediated decrease of *Drs* or *Mtk* induction following *E. faecalis* infection. However, loss of *Xbp1* partially rescued the reduced transcript levels of *Drs* and *Mtk* in the *Sip3 RNAi* flies upon challenging. Regarding fly survival, we observed that the *Xbp1* and *Sip3* double RNAi flies were more resistant to both *E. faecalis* and *S. aureus* than flies with *Sip3 RNAi* alone (**Figures 5C–E**), implying an epistatic relationship between *Xbp1* and *Sip3* in modulating the fly innate immunity.

To obtain additional genetic evidence, we generated *Hrd3*, *Derlin-1*, or *Herp RNAi* flies together with *Xbp1 RNAi*. When these flies were challenged with *E. faecalis*, we obtained similar results with respect to the inductions of AMPs (**Figures 5F, G**). Further, we subjected flies with fat body specific down regulation or ectopic expression of *Xbp1* to bacterial infection. We observed remarkable reduction in the inductions of *Drs* and *Mtk* in the *c564^ts^>Xbp1-EGFP* flies, although it was not the case in the *c564^ts^>Xbp1 RNAi* samples (**Figures 5H, I**). Since Xbp1 mainly functions as a transcription factor governing expression of downstream targets (29, 41–43), it is probably that one/some factor(s) of the Toll pathway is/are under the control of Xbp1. Ultimately, our data point to a working model in which the accumulated Me31B in the ERAD defective flies largely relies on the Xbp1-involved axis of the UPR^ER^ pathway to antagonize the Toll innate immune defense (**Figure 5J**).

## Discussion

ER has been well known for its fundamental importance in the proper post-translational modification and folding of proteins (44). Nevertheless, it is constantly challenged by diverse pathological insults and/or physiological defects, which may lead to ER dysfunction and the accumulation of some misfolded/unfolded proteins in ER (29, 38–40). As an E3 ligase, *Drosophila* Sip3 harbors a critical action in the ubiquitination and degradation of misfolded/unfolded ER proteins (also known as ERAD) (28), thus positively contributing to the maintenance of ER homeostasis to ensure its function. In flies, the Sip3-involved ERAD on retinal degeneration has been well established (28), yet whether it fulfils a role in innate immune modulation remains elusive. In the present study, we observed remarkably reduced induction of the Toll downstream AMPs and increased mortality in the *Sip3* LOF mutants following bacterial infection. Importantly, ectopic expression of *Sip3* in fat bodies almost fully rescued the *Sip3* LOF mutant phenotypes in our experimental system. Further, we provided a series of genetic evidence suggesting that the ERAD is involved in governing the Toll innate immune defense. Our study provides a potential functional linkage between ER homeostasis and innate immunity in *Drosophila.*

How does the ERAD positively contribute to modulating the Toll innate immune defense? Despite our proteomic analyses revealing an increase of a series of proteins/peptides that are primarily involved in several pathways in the *Sip3 RNAi* fat body cells, it appears that none of the crucial factors of the canonical Toll pathway is altered. We therefore shifted our attention unbiasedly to the most increased proteins/peptides (top10) and focused on the proteins shown to be localized in ER, because Sip3 plays a critical role in the ERAD. Between the two potential candidates (Me31B and Tango1 in our study), we identified that Me31B is likely the major downstream effector in the ERAD-mediated Toll innate immune defense, as silencing *Me31B* nearly completely reverses the innate immune defects in the *Sip3*, *Hrd3*, *Derlin-1*, or *Herp RNAi* flies. Nonetheless, our current data cannot address whether the accumulated Me31B is misfolded/unfolded and if so, how the E3 ligase Sip3 promotes its ubiquitination and degradation. It would be worthwhile in the future to obtain a Me31B variant that could somehow mimic its misfolding/unfolding pattern and to inspect its effect in the fly innate immune regulation.

Several lines of evidence have indicated that ERAD and UPR^ER^ can intersect to control ER homeostasis and relevant cellular processes (30, 45, 46). Interestingly, in blocking the Xbp1 axis of the UPR^ER^, we observed a striking impact on the ERAD-involved Toll innate immune reaction, albeit the immune dysfunction in the ERAD-deficient flies cannot be entirely rescued by *Xbp1 RNAi*. One possibility is that the three UPR^ER^ signaling cascades could be somehow reciprocally interrelated under the current experimental conditions. Nevertheless, in accordance with our results, we would like to hypothesize that while the ERAD is blocked, bacterial stimuli possibly lead to excessive accumulation of unfolded/misfolded Me31B protein in ER, where it activates the Xbp1-involved UPR^ER^ to antagonize the Toll innate immune defense in flies (**Figure 5J**). In agreement with this model, we further observed that ectopic expression of Xbp1 in fat bodies markedly prevented the pathogen-driven induction of the Toll downstream AMPs, through a way in which we currently have no knowledge. As a reminder, ERAD has been proposed to be deployed for the clearance of proteins not only misfolded/unfolded (“quality” control), but also excessively abundant (“quantity” control) (47). Indeed, several pioneering studies revealed that Me31B is abundant in variable types of ribonucleoprotein granules as a putative ATP-dependent RNA helicase for post-transcriptionally modulating specific target RNAs (36, 48). It would thus not be surprising if further studies demonstrate a functional utility of Me31B in correlating with and governing the fates of some mRNAs instrumental to the *Drosophila* Toll pathway.

## Materials and methods

### Fly strains

All flies were maintained under normal condition (12h light/12h dark, 65% humidity) with medium (6.65% cornmeal, 7.15% dextrose, 5% yeast, 0.66% agar, 2.2% nipagin, and 3.4 ml/l propionic acid). The *c564-gal4;tub-gal80ts* (*c564^ts^*) was used to allow gene manipulation in fat bodies at adult stage. The following strains were obtained from Bloomington *Drosophila* Stock Center: *Sip3^1^* (#86518), *MyD88^KG03447^* (#14091), *Me31B RNAi* (#38923), *Tango1 RNAi* (#67887), and *UAS-Xbp1-EGFP* (#60730). The following strains were purchased from Vienna *Drosophila* RNAi Center: *Sip3 RNAi 2#* (#6870), *Hrd3 RNAi* (#1161), *Derlin-1 RNAi* (#44210), *Herp RNAi* (#11724), *Xbp1 RNAi* (#109312), *PERK RNAi* (#16427), and *Atf6 RNAi* (#36504). The *Sip3 RNAi 1#* (#TH01506.N) was obtained from Tsinghua Stock Center. The *UAS-Sip3* strain was described previously (49).

### RT-qPCR assay

RT-qPCR assays were performed according to protocols described previously (50). Briefly, whole flies (male) or dissected fat bodies were homogenized in the RNA-easy Isolation Reagent (Vazyme) using glass beads. Total RNA was extracted, followed by cDNA synthesis using the first-strand cDNA synthesis kit (Transgen) according to the manufacturer’s instructions. Quantitative PCR assays were performed using SYBR Green Mix (Transgen) in triplicate on a Bio-Rad iCycler iQ5 PCR Thermal Cycler. All results were normalized to the endogenous reference *RpL32*, which encodes the ribosomal protein Rp49. Primers used in RT-qPCR assays were shown as follows. RpL32-s: GCTAAGCTGTCGCACAAATG; RpL32-as: GTTCGATCCGTAACCGATGT; Drs-s: CGTGAGAACCTTTTCCAATATGATG; Drs-as: TCCCAGGACCACCAGCAT; Mtk-s: CAGTGCTGGCAGAGCCTCAT; Mtk-as: ATAAATTGGACCCGGTCTTG; Dpt-s: TTTGCAGTCCAGGGTCACCA; Dpt-as: CACGAGCCTCCATTCAGTCCAATCTCGG; CecA1-s: ACGCGTTGGTCAGCACACT; CecA1-as: ACATTGGCGGCTTGTTGAG; Xbp1RB-s: CAACCTTGGATCTGCCGCAGGG; Xbp1RB-as: CGCTCCAGCGCCTGTTTCCAG; PERK-s: CTGCGCAGTCTTCGGGACGG; PERK-as: AGCTGCTGAAGGTGCGGCTG.

### Infection, survival rate and bacterial burden assays

Overnight bacterial cultures were harvested and diluted in sterile 1×PBS at a concentration of OD_600 _= 1. Indicated male flies were injected with 4.6nl of diluted bacteria or the same volume of PBS as controls. After infection, flies were immediately transferred to fresh vials (30-40 flies per vial) every day. For survival assays, the numbers of death were counted every day. Flies that died within 2h (< 5% of the total) after bacterial challenge were not considered in the analyses.

For bacterial burden assays, 10 flies were harvested, dipped in 75% EtOH and subsequently volatilized with EtOH on the fly pad for several minutes. Flies were then homogenized in 200μl of sterile 1×PBS. The ground homogenate was serially diluted and 100μl of each diluent was placed on LB agar. Plates were incubated in a 30°C culture hood overnight before counting the numbers of bacterial colonies.

### Western blot assay

Western blot assays were performed as described previously (51). In brief, fat bodies were lysed in lysis buffer (150mM NaCl, 50mM Tris-HCl, pH=7.4, 10% glycerol, 0.5% TritonX-100, and 1mM PMSF). Lysates were then centrifuged at 12,000rpm for 10min at 4°C. The supernatant was subjected to Western blot analysis. The Sip3 polyclonal antibody was generated by immunizing mice with purified GST-tagged Sip3^527-626^. The β-Tubulin antibody was purchased from Cowin (1:2000, Cat#CW0098M).

### Proteomic analysis by LC-MS/MS

Proteomic analysis by LC-MS/MS was performed according to previously described method (25). Briefly, Fat bodies were harvested from indicated flies in three independent biological replicates, followed by lysate preparation using lysis buffer (50mM Tris-HCl, pH=7.5, 150mM NaCl, 0.5% TritonX-100, 10% glycerol with 1mM PMSF). Samples were precipitated overnight with acetone on ice. Proteins/peptides were digested with Trypsin (Promega) and then desalted (Piecrce^TMC^-18 Spin Columns, Thermo) according to manufacturer’s instructions. Peptides were then subjected to LC-MS/MS assays. Resulting MS/MS data were processed using Thermo Proteome Discovery (version 1.4.1.14) and tandem mass spectra were searched against UniProt-*Drosophila* database.

### Statistical analysis

All statistical analyses were performed by using GraphPad Prism 8. Data were shown as mean and standard errors. Statistical significance was determined by using the ANOVA or Mann-Whitney tests except for survival assays, in which the Log-Rank test (Kaplan-Meier method) was used for statistical analysis. The p value of less than 0.05 was considered statistically significant. *p < 0.05; **p < 0.01; ***p < 0.001; ns, not significant.

## Data availability statement

The data presented in the study are deposited in the "Mendeley" repository (https://data.mendeley.com/datasets/6xbrt3n75v/1).

## Author contributions

YZ, LL, QC, and SJ conceived and designed the experiments. YZ, LL, ChuZ, ChaZ, TH, RD, YJ, KS, YX, and QC performed the experiments. YZ, LL, and QC analyzed the data. YZ and LL performed statistical analyses. YZ, AG, QC, and SJ wrote the manuscript. All authors contributed to the article and approved the submitted version.
