# Supplementary material for: Endoplasmic reticulum-associated protein degradation contributes to Toll innate immune defense in Drosophila melanogaster
Source: Front Immunol. 2023 Jan 19;13:1099637. doi: 10.3389/fimmu.2022.1099637 (PMC9893508; doi:10.3389/fimmu.2022.1099637)
Supplement: Supplementary file 1 [file DataSheet_1.pdf]

Figure S1

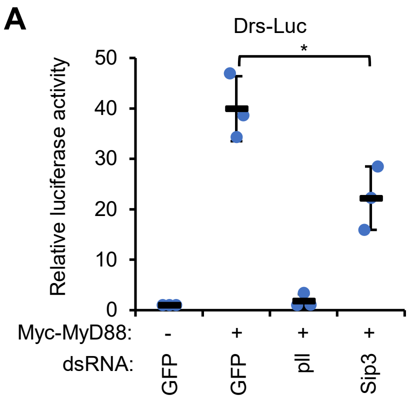

Supplementary Figure 1. Knockdown of *Sip3* decreases the Myd88-driven Drs-Luc activity in *Drosophila* S2 cells.
(A) S2 cells were treated with various dsRNAs for 48 h (*GFP* dsRNA for the first and second columns; *pll* dsRNA for the third column as positive control; *Sip3* dsRNA for the last column), followed by transfection with different combinations of expressing plasmids as indicated. 36 h post transfection, cells were lysed for dual-luciferase assays. Each dot represents one independent replicate. Data were shown as mean ± standard errors. *$p<0.05$.

Figure S2

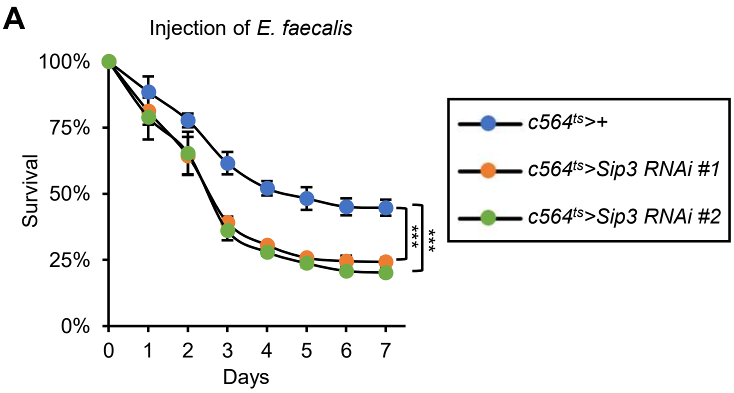

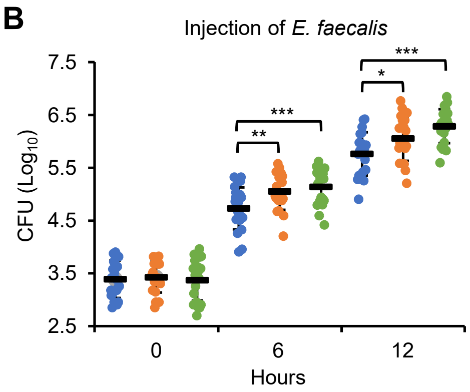

Supplementary Figure 2. *Sip3* modulates host anti-microbial defense.
(A) Flies including *c564ts>+, c564ts>Sip3 RNAi #1, and c564ts>Sip3 RNAi #2* were infected with *E. faecalis*. Flies were then counted for mortality every day. The numbers of flies were as follows. *c564ts>+*: 105, 106, 104; *c564ts>Sip3 RNAi #1*: 103, 105, 106; *c564ts>Sip3 RNAi #2*: 103, 102, 103. Data were shown as mean ± standard errors. ***p<0.001.
(B) Flies including *c564ts>+, c564ts>Sip3 RNAi #1, and c564ts>Sip3 RNAi #2* were infected with *E. faecalis*. At indicated time points (0, 6, and 12 h), flies were subjected to bacterial burden assays. Each dot represents one independent replicate (10 flies for each replicate). Data were shown as mean ± standard errors. *p<0.05; **p<0.01; ***p<0.001.

Figure S3

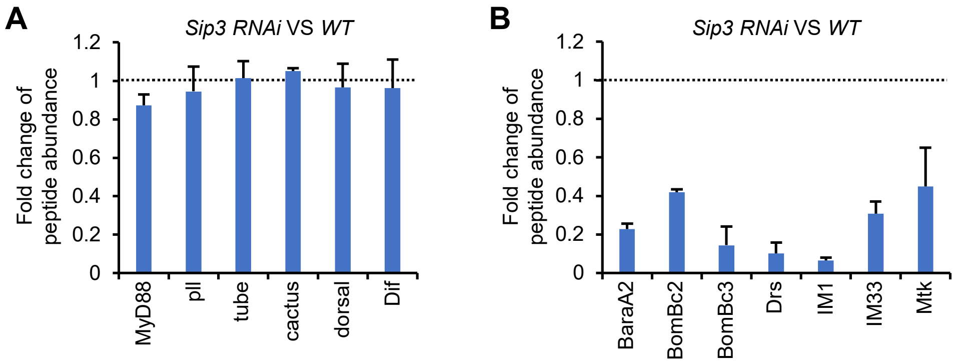

Supplementary Figure 3. Toll downstream AMPs are decreased in *Sip3 RNAi* flies compared to those in the controls.
(A and B) Fold changes of the key factors (A) and the AMPs (B) of the Toll pathway were analyzed and shown based upon the proteomic assay. Data were shown as mean ± standard errors.

Figure S4

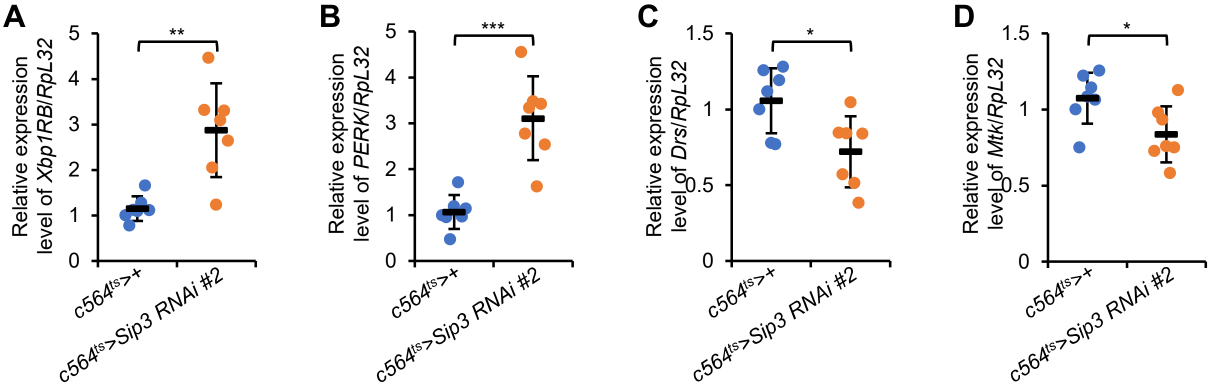

Supplementary Figure 4. UPR^{ER} is activated in the *Sip3 RNAi* fat bodies.

(A-D) Fat bodies were dissected from flies including *c564^{ts}>+* and *c564^{ts}>Sip3 RNAi #2*, followed by RT-qPCR assays to monitor the expression levels of *Xbp1RB* (A), *PERK* (B), *Drs* (C), or *Mtk* (D). Each dot represents one independent replicate (10 flies for each replicate). Data were shown as mean ± standard errors. *p<0.05; **p<0.01; ***p<0.001.
